# Subdural Hematoma: An Adverse Event of Electroconvulsive Therapy—Case Report and Literature Review

**DOI:** 10.1155/2012/585303

**Published:** 2012-11-14

**Authors:** Ranganath R. Kulkarni, Sateesh Melkundi

**Affiliations:** ^1^Department of Psychiatry, SDM College of Medical Sciences and Hospital, Karnataka, Dharwad 580009, India; ^2^Department of Neurosurgery, SDM College of Medical Sciences and Hospital, Karnataka, Dharwad 580009, India

## Abstract

Electroconvulsive therapy (ECT) is commonly used in the management of medication nonresponsive depressive disorder, with proven efficacy in psychiatric practice since many decades. A rare complication of intracranial bleed following this therapeutic procedure has been reported in sporadic case reports in the English literature. We report a case of such a complication in a 42-year-old male, a known case of nonorganic medication nonresponsive depressive disorder for the last two years who required ECT application. Presenting symptoms included altered mental state, urinary incontinence, and repeated episodes of vomiting; following ECT procedure with magnetic resonance imaging (MRI) of the brain suggestive of bilateral acute subdural hematoma. Despite the view that it may be used in neurological conditions without raised intracranial tension, it will be worthwhile to be vigilant during post-ECT recovery for any emergent complications.

## 1. Introduction

Electroconvulsive therapy (ECT) is a treatment modality in which electricity is used to create a seizure in a patient who has received general anaesthesia. ECT is most commonly used to treat depressive disorder and has been shown to be effective for many such patients with suicidal ideations, as also they do not respond to medication trials or psychotherapies [1, 2]. Recently two case reports of chronic subdural haematoma following modified ECT were described [3, 4]. Surprisingly, another case report of bipolar disorder with traumatic acute subdural hematoma being treated with series of ECT a week following cranioplasty is also known [5]. Recent large outcome studies have reported no cases of cerebral haemorrhage [6, 7]; nevertheless emerging case reports in the past decade may notify some of the rarest complications associated with modified ECT, cause for which remains obscure. In spite of the frequent usage of ECT only few serious complications have been reported in the English literature [3, 4, 8]. This is particularly true of the intracranial bleed, which is sporadically described and reported.

## 2. Case Report

A 42-year-old married adult male with right hand dominance, from urban and upper socioeconomic background presented with gradual onset, nonprogressive, pervasive depressed mood of two-year duration with symptoms of insomnia, anorexia, lack of interest and enjoyment, and ideas of worthlessness, hopelessness, and helplessness, leading to sociooccupational dysfunction. He also presented with suicidal ideations of two-week duration, with Hamilton Depression Rating Scale 17-item (HAMD-17) [9] score of 25 (very severe depression) at the time of admission. He was diagnosed as having chronic depressive disorder as per ICD-10 diagnostic criteria [10]. He had been on treatment for a year from a psychiatrist on escitalopram (20 mg/day), mirtazapine (45 mg/day) and clonazepam (0.5 mg/day), but showed no significant clinical improvement. He had no prior medical history of hypertension, diabetes mellitus, fall/head injury or anticoagulant/antiplatelet drug intake, bleeding diatheses, renal problems, epilepsy, or alcoholism. Patient had cordial family and work atmosphere with no family history of psychiatric illnesses. His vital parameters, physical examination, fundoscopy, and neurological opinion revealed none of the problems which can be attributed to organic brain pathology. Biochemical and haematological investigations like blood glucose, coagulation profile, liver function tests, thyroid profile, and complete blood counts were within normal limits. 

**Figure 1:**
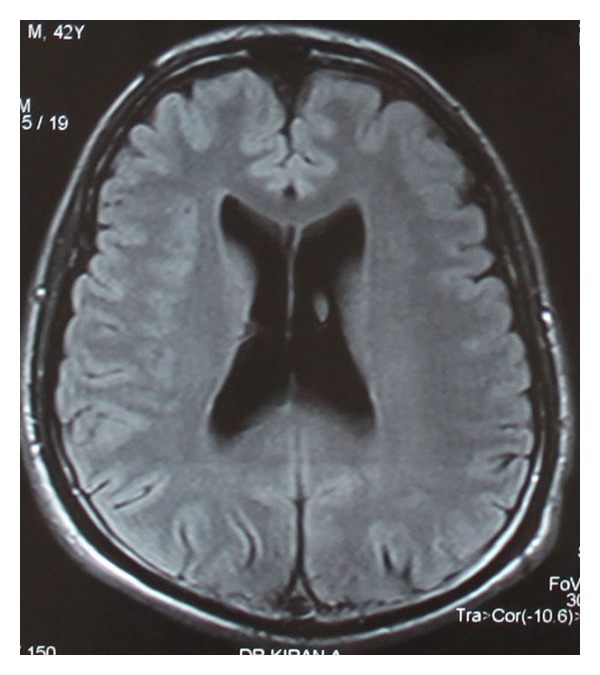
Pre-ECT cranial magnetic resonance imaging with contrast done to rule out organic causes of depression showing normal study.

**Figure 2:**
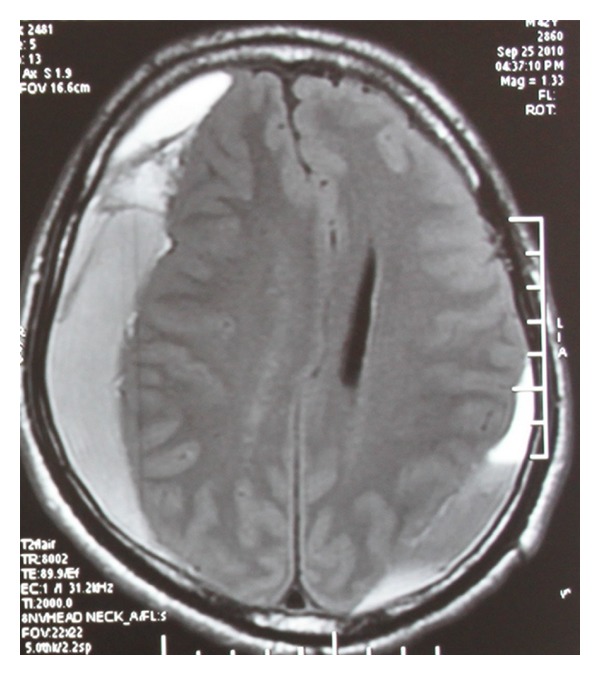
Post-ECT cranial noncontrast magnetic resonance imaging showing bilateral asymmetric extensive acute subdural hematoma extending over right frontoparietal and left parietal areas with mass effect and midline shift to left.

In view of presence of chronic severe depression with recent suicidal ideations, poor response to treatment with antidepressants, and absence of psychosocial stressors, patient underwent magnetic resonance imaging (MRI) brain screening to rule out organic causes for depression (Figure 1), prior to the consideration of ECT. Standard protocol as prescribed by the Royal College of Psychiatrists for ECT was followed [11]. Written informed consent for the procedure was taken from the patient and caregiver. His vital parameters were 124/80 mm of Hg of blood pressure with pulse rate of 80 beats per minute. During the first sitting of modified ECT, patient received 0.6 mg glycopyrrolate, 80 mg propofol, and 50 mg succinyl choline. ECT was delivered using standard brief pulse ECT machine with bitemporal electrode placement and delivery of brief pulse waveform electrical stimulus strength of 120 mC dosage, 1.5 msec pulse width, 800 mA pulse amplitude, and 125 pulse per second for duration of 1.2 seconds resulting in an adequate motor seizure duration of 47 seconds. During ECT procedure, patient had rise in blood pressure to 158/96 mm of Hg with pulse rate of 110 beats per minute, without return to normal values after the procedure. Post-ECT recovery was delayed as patient had altered mental state, repeated episodes of vomiting and bladder incontinence with Glasgow Coma Scale (GCS) [12] score of E2 M2 V4 at the end of one hour after ECT. Bilateral pupils were middilated but reactive to light. His fundoscopy showed papilledema suggestive of raised intracranial tension. Patient did not sustain any fall or head injury prior to, during, or soon after the procedure. A cranial MRI reported bilateral asymmetric (right more than left side) extensive acute subdural hematoma over right frontoparietal and left parietal areas with mass effect and midline shift to left side (Figure 2). Overlying bone window was normal. Patient underwent an emergency neurosurgical intervention, and hematoma was evacuated with burr-hole operation. All psychiatric medications were discontinued until surgically stabilized. After 24 hours of evacuation, patient had Mini-Mental Status Examination (MMSE) [13] score of 28 out of 30, and GCS score improved to E4 M6 V5. After 72 hours, patient's condition deteriorated due to the recollection of bilateral acute on subacute subdural hematoma with mass effect, confirmed by cranial MRI study. Neurosurgical reexploration and reevacuation stabilized the patient's condition without any further recollection of blood in subdural space. A week later, patient had moderate depression without suicidal ideations, with a significant decrease in HAMD-17 item scores from preoperative score of 25 to 14. Escitalopram was not considered in view of risk of bleeding with selective-serotonin reuptake inhibitors, and the patient was discharged with mirtazapine (15 mg/day). On subsequent followup, patient showed improvement with mirtazapine (30 mg/day).

## 3. Discussion

ECT is an accepted treatment modality for severe psychiatric illnesses since many decades. It is considered as an effective treatment for medication nonresponsive severe depressive disorder and a life-saving therapeutic procedure in those with suicidal ideations as was the case in our patient. Risks and adverse events of ECT can be divided into two categories: firstly, those medical complications that can be substantially reduced by the use of appropriately trained staff, best equipment, and best methods of administration; secondly, those side effects, such as transient posttreatment confusion, headache, and spotty but persistent memory loss that can be expected even when an optimal treatment approach is used. In the recent series reported, there were 2.9 deaths per 10000 patients; another series reported 4.5 deaths per 100000 treatments. Overall, the risk is not different from that associated with the use of short-acting barbiturate anaesthetics. In another study of almost 25000 treatments, a complication rate of 1 per 1300 to 1400 treatments was found [2]. These included laryngospasm, circulatory insufficiency, tooth damage, vertebral compression fractures, status epilepticus, peripheral nerve palsy, skin burns, and prolonged apnea. The time it takes to recover clear consciousness may vary from minutes to several hours, depending on individual differences in response, the type of ECT administration, the anaesthetic substance utilised, the spacing and number of treatments given, and the age of the patient [2]. Advanced medical technology has substantially reduced the complications associated with ECT.

Intracranial parenchymal bleeds form an important subgroup of extremely serious although very rare complication, which has been scantily reported following modified ECT. It is particularly difficult to establish the iatrogenic cause of intracranial bleed. Nevertheless, the true incidence of intracranial bleeds after ECT seems to be underestimated. However, cases of neurological insults such as subdural bleed were not found to increase or deteriorate during an ECT with observed safety in these patients [14, 15]. In patients with neurological abnormality after ECT, it may be more difficult than usual to diagnose an intracranial insult such as intracranial bleed. However, any persistent change in the level of sensorium, additional focal neurological signs, epileptic convulsions, repeated episodes of vomiting, and incontinence in patient receiving ECT should arise a highest degree of suspicion of intracranial adverse events that justify the need for neuroimaging investigations. Computed tomography (CT) or MRI scans are valuable in diagnosing and localising intracranial insults as was in our case. Awareness of possibility of such potential adverse event among treating psychiatrists, after ECT and in followup of a case having undergone ECT, results in lifesaving of such patients.

Acute, rapidly evolving subdural hematomas are due to tearing of bridging pial veins, and symptoms are caused by compression of the brain by an expanding clot of fresh blood. ECT has been administered in psychiatric patients with neurological conditions without raised intracranial tension, including postcraniotomy, cranioplasty, subdural hematomas, and brain tumours in the literature [5]. In our case, acute subdural hematoma presented in temporal association with ECT and hence possibly could be secondary to ECT. The exact mechanism of such causation remains obscure. However, possible explanations for this rare complication are put forth. Firstly, anterior communicating artery or MCA bifurcation berry aneurysms may rupture into the adjacent brain or subdural space and form a hematoma large enough to produce mass effect [16]. Secondly, subdural hematoma can also result as a potentially serious consequence of spontaneous intracranial hypotension (SIH) with reported incidence of 10% [17, 18]. The diagnosis of SIH should be considered in patients presenting with subdural hematoma in the absence of trauma and with normal clotting, particularly as subdural hematomas secondary to intracranial hypotension may recur following drainage, and treatment of the underlying cause is required [19].

It must be noted that hyperventilation done prior to ECT reduces CSF pressure and may therefore contribute to the reduction in intracranial pressure. In general it can be stated that changes in cerebral blood flow always induce changes in the cerebral blood pressure in the same direction. Certain cases of arteriosclerosis with decreased cerebral blood flow show reduced CSF pressure, which may reflect the reduction in size of the intracranial vascular bed. The reduced intracranial circulation may also curtail CSF production and lead to intracranial hypotension [20]. 

## 4. Conclusion

Application of ECT, although a proven efficacious and safe therapeutic procedure in psychiatric practice since many decades, should be administered and monitored during and after each treatment for any adverse event. A cranial CT or MRI may be done in suspected organic causes of psychiatric disorders to rule out any intracranial pathology before considering ECT, especially in cases of refractory depression. Despite the view that it may be used in severe psychiatric illnesses with neurological conditions without raised intracranial tension [5], recent case reports of chronic subdural haematoma following modified ECT [3, 4] emphasize the need to be vigilant, especially in case of delayed recovery, persistent delirium, or signs of organicity (altered sensorium, vomiting, and incontinence) following ECT for any emergent complications such as subdural hematoma.
